# Wormhole formation in fluid-driven granular flow

**DOI:** 10.1038/s42005-025-02366-w

**Published:** 2025-11-24

**Authors:** Miles L. Morgan, David W. James, Martin Monloubou, Bjørnar Sandnes

**Affiliations:** 1 Complex Fluids Research Group, Department of Chemical Engineering, Swansea University, Swansea, UK; 2 ENSTA Bretagne, UMR CNRS 6027, IRDL, Brest, France

**Keywords:** Fluid dynamics, Applied physics, Soft materials

## Abstract

Fluid-driven flow of granular material leads to complex behaviour and emergent instabilities in many natural and industrial settings. However, the effect of using fluid flow to vertically drive a dense bed of sedimenting grains is not well documented. Here we find contrasting behaviours in a submerged fluid-driven silo, including fingering patterns, porous flow, classical silo flow, and the formation of straight, semi-dilute wormhole-like channels. Once formed, these channels rapidly propagate towards the outlet and act as a bypass of the wider packing. The onset of this instability occurs when the gravity-driven grain flow at the free surface is insufficient to supply the fluid-assisted central region below the interface. Balancing empirical models of these flows predicts the height at which channels emerge as a function of grain size and flow rate. These findings provide a framework for predicting and controlling fluid-grain interactions in natural hazards, industrial processing, and geophysical flows.

## Abstract (web summary)

Granular materials exhibit complex behaviors in various flow regimes, with fluid-driven dynamics leading to phenomena like erosion and pattern formation. Here, the authors experimentally investigate flow regimes in a submerged quasi-two-dimensional fluid-driven silo, demonstrating wormhole-like instabilities and providing insights into how gravitational and viscous effects dictate the observed instabilities.

## Introduction

Granular materials are known to exhibit complex behaviours in flow across inertial, collisional, quasistatic and viscous regimes^1,2^, and even superficially simple geometries such as silo flow still receive significant attention^3–6^. When driven by fluid, grains are subjected to hydrodynamic forces which can lead to erosion and deposition, porous flow and pattern formation^7–9^, with gas driven into granular packings inducing fracturing^10^ and Saffman-Taylor-like finger instabilities at the grain interface^11^.

The granular flow rate in a classical dry silo is described by the Beverloo equation^12^, chiefly depending on the size of the outlet and grain characteristics, while internally, the velocity of grains converging towards the outlet is often described through a diffusion framework that conveniently captures the Gaussian velocity profiles as they spread upwards through the silo^13–15^. This centralised flow causes a depression at the upper grain boundary; the slopes eventually exceed the angle of movement, resulting in avalanching at the free surface and the formation of a pointed V-shaped interface^6,16^. Meanwhile, the imposition of fluid flow through a silo can lead to the control of grain flow rate beyond Beverloo’s base law^4,17^, with the diffusion in the velocity field also affected^18^. However, the effects of imposed fluid flow on the grain interface of a fluid-driven silo are not well understood. Flow in such conditions is not just of interest for the efficient hydraulic transport of granular materials, but also regarding natural instances of fluid-driven non-buoyant granular media in confined environments that are difficult to observe, such as suffosion in sink hole formation^19^, and erosion in fractures^20^ and fault gouge that can weaken faults^21,22^.

Here we experimentally demonstrate and characterise regimes of flow in a submerged quasi-two-dimensional fluid-driven silo. Grains of various diameters *d* are drained with fluid from a vertical Hele-Shaw cell of fixed plate spacing and outlet size, at a controlled flow rate *Q*. We find “wormhole”-like instabilities occurring with all grain sizes at a range of flow rates, in addition to finger-like instabilities and behaviour similar to classical silo flow. The gravitational avalanche flow down the sides of the V-shaped depression replenishes mass to the central parts of the silo and acts to stabilise the interface at fluid flow rates that would otherwise induce viscous finger instabilities in a horizontal cell. However, observations suggest that when a critical flow rate is exceeded, wormholes form due to an imbalance between the stabilising avalanche-like surface flow and the grain flow drawn by the central region of the silo below.

## Results

**Fig. 1:**
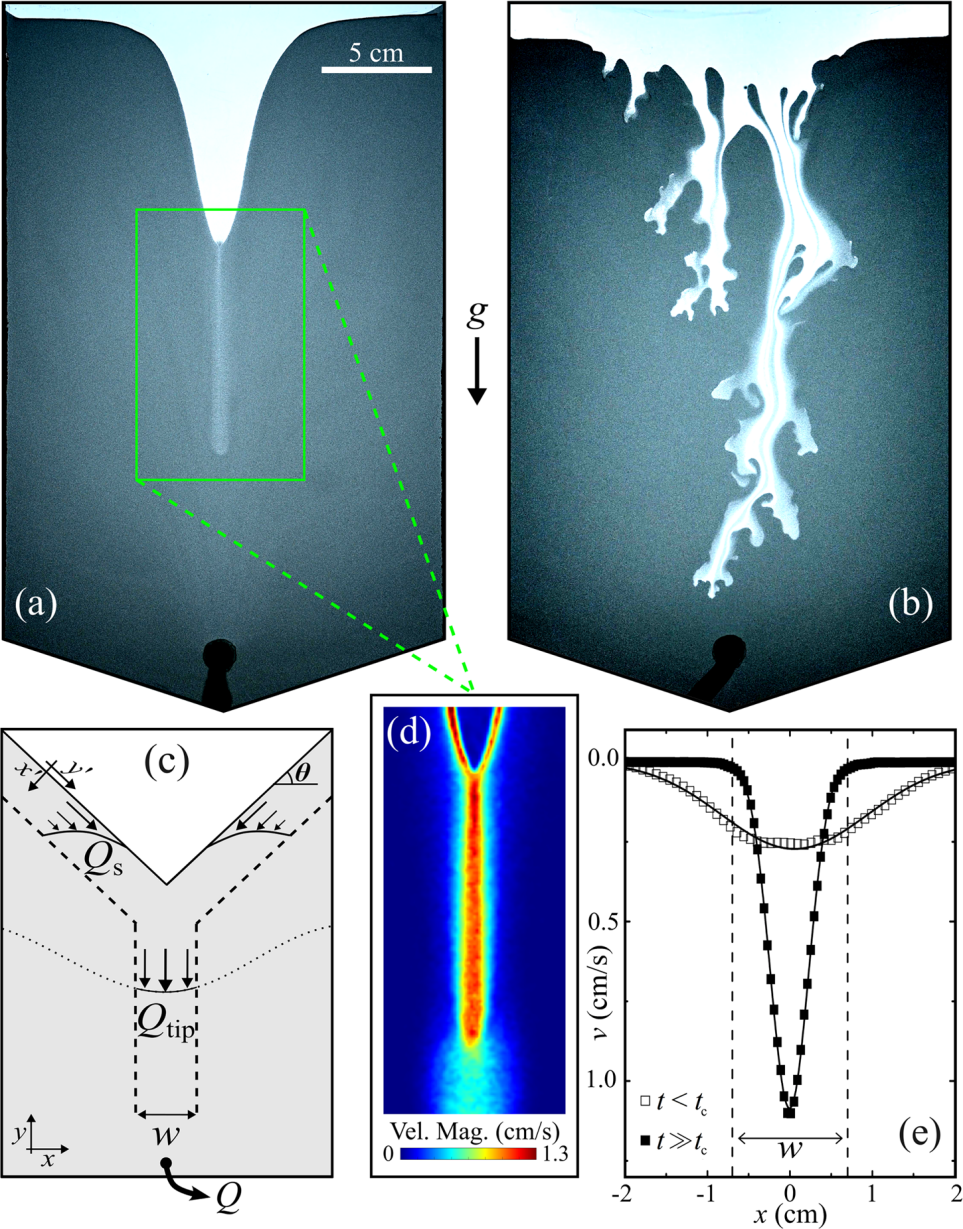
Instabilities in a fluid-driven silo. **a** A wormhole advancing in fluid-driven silo flow at a total flow rate of 3 ml/min and **b** viscous fingers at 50 ml/min for 75-100 μm grains. Flow is controlled by a syringe pump that withdraws material from an outlet located at the base, which is visible as a black region in the centre at the bottom of the image. **c**–**e** illustrate wormhole characteristics. **c** Schematic of falling free surface flow *Q*_s_ feeding a central silo region of width *w* and flow rate *Q*_tip_ before wormhole formation, with a total flow rate *Q* withdrawn from the outlet. **d** Velocity magnitude of the wormhole region measured with particle image velocimetry and represented as displayed in the colourbar. **e** Vertical grain velocity *v* as a function of horizontal distance from the silo centre *x* at a time *t* before the wormhole formation time *t*_c_ (open squares), and after *t*_c_ (closed squares) at 1.75 ml/min ~5 cm above the outlet, both fitted with Gaussian functions. The width of the central silo region is marked as *w*.

**Fig. 2:**
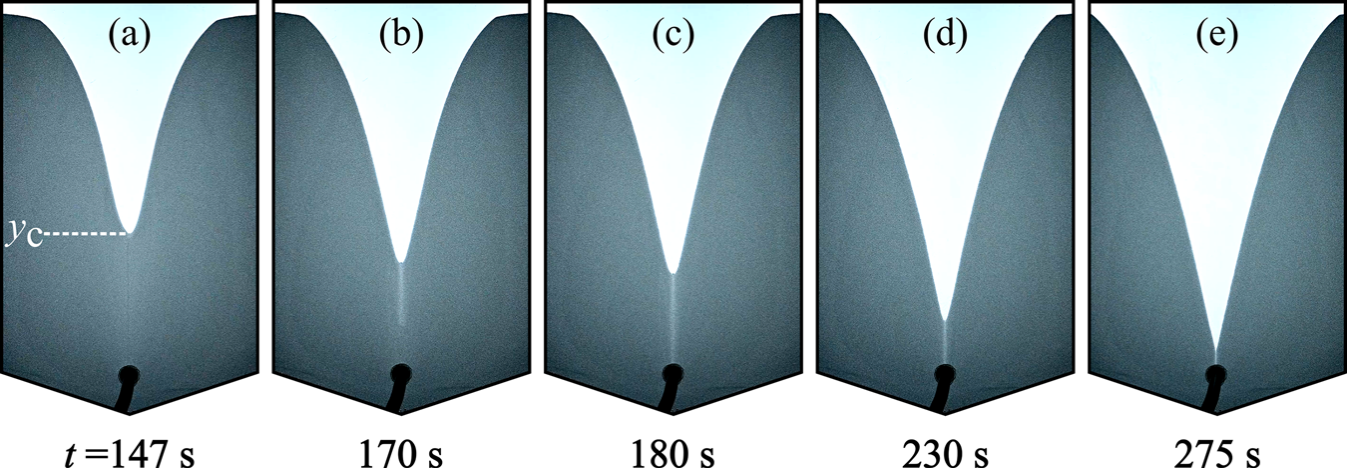
Time evolution of wormhole progression. **a** The silo is depleted such that the height of the grain boundary reaches the critical wormhole formation height *y*_c_, triggering wormhole formation. **b** The wormhole rapidly descends, reaching the outlet in (**c**). **d** The wormhole remains stable while the silo empties, until the grain boundary converges with the outlet in (**e**). Video for this experiment is provided in Supplementary Movie 1.

Four principle regimes of flowing behaviour were observed in experiments. (i) At low total flow rate *Q* the granular bed remains static with Darcy flow of water through the pore space. (ii) Above a critical *Q* the grains in the outlet are mobilised, leading to stable silo flow. Increasing *Q* further leads to fluid-driven instabilities: (iii) wormholes and (iv) viscous fingers, shown in Fig. 1a, b respectively. Video examples of these are presented in Supplementary Movie 1 and Supplementary Movie 2. In cases of wormhole formation, initial flow of the granular bed typically is similar to classical silo flow, with an internal velocity field that spreads outwards with height resulting in a depression at the centre of the upper grain boundary, causing avalanching at the free surface and a V-shaped grain-fluid interface. However, as the grain boundary descends, at some height *y*_c_ above the outlet, a relatively dilute, narrow channel emerges at its centre, penetrating the granular packing, advancing directly towards the outlet significantly faster than the remaining grain-fluid interface. Figure 1d shows grain velocity magnitude measured by PIV during wormhole growth. Within the wormhole, the grain velocity is several times that of the wider flowing, dense packing otherwise exhibited in the silo, as illustrated in Fig. 1e which plots vertical grain velocity *v* before and after wormhole formation. As the wormhole approaches the outlet, the wider Gaussian velocity profiles typical of silo flows cease, with all grain flow above the wormhole tip occurring in the newly formed central channel, which incidentally is itself described well with a Gaussian function. The formation and growth of a wormhole with time for a typical case is shown in Fig. 2. Once the wormhole tip reaches the outlet, all grain flow in the silo takes place within and around the wormhole, which now spans fully from the top of the granular layer to the outlet. The wormhole remains stable as the silo empties until the grain boundary converges with the outlet. Such channelisation is comparable to dissolution wormholes in porous media, that effectively act as a bypass for injected fluid^23^.

**Fig. 3:**
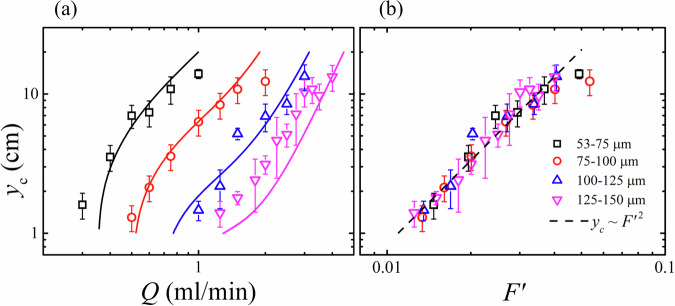
Location of wormhole onset. **a** Wormhole formation height *y*_c_ as a function of total flow rate *Q* for all grain sizes. Solid lines represent the height *y*_c_, solved numerically through Eqs. (3), (5) with ${Q}_{{{{{\rm{s}}}}}}^{* }={Q}_{{{{{\rm{tip}}}}}}$. Colours and symbols indicate different grain size intervals. **b** *y*_c_ plotted as a function of the dimensionless number ${F}^{{\prime} }$ defined in Eq. (1) that quantifies viscous and gravitational effects. The dashed line represents a power law fit of index 2. Error bars represent the standard deviation of repeat measurements.

Wormhole formation occurs higher up in the silo—and therefore sooner relative to depleted silo volume—as flow rate *Q* is increased, and as grain size *d* is decreased. This critical formation height *y*_c_ is plotted as a function of *Q* in Fig. 3a for each grain size. For a general understanding of the mechanisms causing this behaviour, we may first parametrise the influence of viscous and gravitational effects in the silo with the dimensionless number

1 $${F}^{{\prime} }=\frac{18\eta Q}{\Delta \rho g{d}^{2}A}$$

where *Δρ* is the density difference between the solid and liquid phase, and *η* is fluid viscosity and *A* is silo cross sectional area. This accommodates the independent variables and is equivalent to the ratio of the settling velocity of a grain in fluid and the superficial velocity in the silo imposed by the controlled flow rate. ${F}^{{\prime} }$ has previously been used to quantify the changing velocity field within such a fluid-driven silo^18^. Figure 3b displays wormhole formation height as a function of ${F}^{{\prime} }$, finding a collapse for all grain sizes, and an approximate relation ${y}_{{{{{\rm{c}}}}}} \sim {F}^{{\prime} 2}$, suggesting as viscous forces become more influential with respect to gravity, wormholes form more easily during silo depletion. The scaling may be broadly justified by approximating the width of the silo’s flowing region to increase parabolically with height, as described by the normal diffusion model in classical silo flow^13^. We can consider a local ${F}^{{\prime} }(y)=\frac{18\eta v(y)}{\Delta \rho g{d}^{2}}$, where *v*(*y*) is the magnitude of the vertical grain velocity as a function of height *y* in the silo. The parabolic approximation leads to the scaling of this velocity $v(y) \sim Q/\sqrt{dy}$ in terms of the presently relevant variables^18^. If a wormhole forms when the grain interface reaches a height *y*_c_ and there exists some corresponding critical local ${F}^{{\prime} }({y}_{{{{{\rm{c}}}}}})$ - where the viscous effects are sufficiently dominant over gravity—the height at which it occurs therefore scales as ${y}_{{{{{\rm{c}}}}}} \sim {(\eta Q/\Delta \rho g{d}^{5/2})}^{2} \sim {F}^{{\prime} 2}/d$ for a given silo. This generally captures the behaviour of Fig. 3, with the exception of a greater *d* dependence, which may be attributed to the approximation made regarding the silo velocity field, and the generality of ${F}^{{\prime} }$, that merely considers the drag acting on a single sphere.

In order to understand the onset of this instability in more detail, we will consider the behaviour around the central tip of the interface, and propose that it occurs due to an imbalance between the grain flow avalanching towards the centre, and that which is drawn by the silo below.

### Surface avalanche flow

**Fig. 4:**
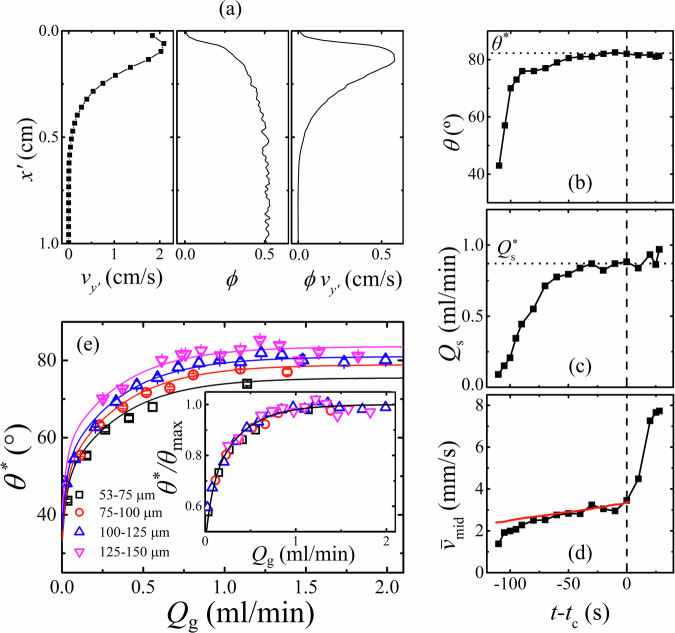
Surface grain flow measurements. **a** Measured local surface grain velocity ${v}_{{y}^{{\prime} }}$, and solid fraction *ϕ* with calculated local grain flux $\phi {v}_{{y}^{{\prime} }}$ in the steady state for 125–150 μm grains at a total flow rate of 2.5 ml/min, plotted as a function of depth perpendicular to the surface flow ${x}^{{\prime} }$. **b** Free surface angle *θ*, **c** instantaneous surface flow rate *Q*_s_, and **d** average central silo velocity ${\bar{v}}_{{{{{\rm{mid}}}}}}$ 1 cm below the surface, plotted as a function of time from wormhole formation *t* − *t*_c_. Vertical dashed lines represent the start of wormhole formation at *t* = *t*_c_. The red solid line represents average velocity in the central region of the silo 1 cm below the surface according to Eq. (5). **e** Steady state free surface angle θ^*^ as a function of silo grain flow rate *Q*_g_ for each grain size fitted with Eq. (2). Inset: θ^*^ normalised by ${\theta }_{\max }^{* }$ and fitted with Eq. (2) with the approximation ${\theta }_{{{{{\rm{r}}}}}}/{\theta }_{\max }^{* }\approx 0.45$. Colours and symbols indicate different grain size intervals; error bars represent the standard deviation of multiple angle measurements.

Unlike dry silos where the grain flow rate is typically low and the free surface flow is sufficient to maintain a steady interface near the angle of repose^16^, fluid-driven silos are capable of high grain flow rates within the silo, while surface flow in such submerged systems is inhibited by viscous forces^24,25^. The avalanching surface grain flow observed presently is similar to both dry and submerged heap and avalanche surface flows, being most rapid and dilute near the free surface, whilst becoming dense with decaying velocity below (see Fig. 4a)^24,26^. Figure 4b displays the free surface angle *θ* measured at the steepest section of the grain surface, where there exists a region of constant slope. Plotted as a function of time leading up to the point of wormhole formation *t* − *t*_c_ for a typical case, *θ* undergoes an initial increase when the V-shaped depression first forms, before becoming a constant dynamic angle of repose θ^*^ with time. The steepening angle accommodates a larger avalanche flow rate, shown in Fig. 4c, which also reaches a plateau ${Q}_{{{{{\rm{s}}}}}}^{* }$. Increasing imposed total flow rate *Q* through the system results in a higher grain flow rate within the silo *Q*_g_. To balance this flow at the surface, θ^*^ increases, shown in Fig. 4e, but approaches a maximum value ${\theta }_{\max }^{* }$ which is observed to increase with grain size, as determined by curve fitting and listed in Table 1. In fact, the angles observed here are very high (up to 80°) and exceed that of superstable dry heap flow reported in thin channels^27,28^. These high inclination angles are enabled by friction exerted at the sidewalls that retards the flow, necessitating a steeper slope to sustain a given flux. To describe θ^*^ empirically, a two-phase exponential association is employed that considers the static angle of repose *θ*_r_ and maximum dynamic angle ${\theta }_{\max }^{* }$:

2 $${\theta }^{* }={\theta }_{{{{{\rm{r}}}}}}+\left({\theta }_{\max }^{* }-{\theta }_{{{{{\rm{r}}}}}}\right)\left[p\left(1-{e}^{-\frac{{Q}_{g}}{{q}_{1}}}\right)+\left(1-p\right)\left(1-{e}^{-\frac{{Q}_{g}}{{q}_{2}}}\right)\right]$$

The numerical coefficient *p* = 0.36 weights the respective decay constants *q*_1_ = 0.072 ml/min and *q*_2_ = 0.35 ml/min that describe the curve from *θ*_r_ at no flow to the ${\theta }_{\max }^{* }$ plateau. The inset of Fig. 4e consists of the same data normalised with ${\theta }_{\max }^{* }$. The collapse reflects the ratio ${\theta }_{{{{{\rm{r}}}}}}/{\theta }_{\max }^{* }\approx 0.45$ that approximately holds for all grain sizes.

**Table 1:** Physical characteristics of grain samples

Sieve range (μm)	*d* (μm)	*θ*_r_ (°)	${\theta }_{\max }^{* }$(°)	*ϕ*_s_
53–75	64.5	32.5 ± 0.1	75.5 ± 1.1	0.553
75–100	87.3	33.2 ± 0.9	78.9 ± 0.3	0.573
100–125	122.8	35.9 ± 0.2	81.0 ± 0.7	0.559
125–150	142.7	40.6 ± 1.4	83.6 ± 0.5	0.546

Granular materials were sieved within various size ranges and their properties quantified: average grain diameter *d*, in situ static angle of repose *θ*_r_, maximum dynamic angle of repose ${\theta }_{\max }^{* }$ and static solid fraction *ϕ*_s_ measured in the silo.

**Fig. 5:**
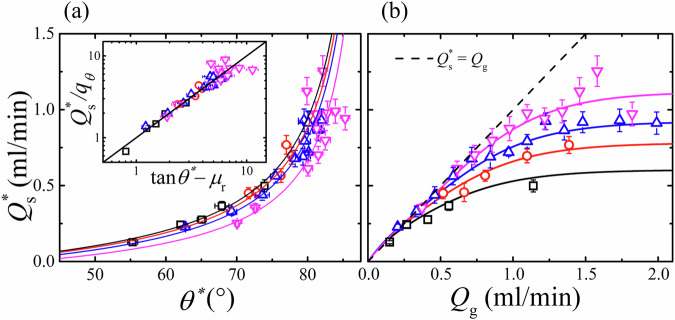
Surface grain flow rate. **a** Steady state free surface flow rate ${Q}_{{{{{\rm{s}}}}}}^{* }$ plotted as a function of angle θ^*^ and fitted with Eq. (3). Inset: ${Q}_{{{{{\rm{s}}}}}}^{* }$ normalised by surface flow rate fitting parameter *q*_*θ*_ plotted as function of $\tan {\theta }^{* }-{\mu }_{{{{{\rm{r}}}}}}$ and fitted with a rearranged Eq. (3), where *μ*_r_ is the effective friction coefficient. **b** ${Q}_{{{{{\rm{s}}}}}}^{* }$ as a function of total grain flow rate *Q*_g_. Solid lines represent the combination of Eqs. (2), (3); colours and symbols indicate different grain size intervals; error bars represent the standard deviation from repeat measurements.

Figure 5a shows the strong dependence of steady state surface flow rate ${Q}_{{{{{\rm{s}}}}}}^{* }$ with angle θ^*^. Data for each grain size is fitted with the function

3 $${Q}_{{{{{\rm{s}}}}}}^{* }={q}_{\theta }(\tan {\theta }^{* }-{\mu }_{{{{{\rm{r}}}}}})$$

reflecting similar non-linear behaviour in immersed avalanches^24^ and qualitative similarity to dry super stable heap flow^28^. Here, the static limit is parametrised by an effective friction ${\mu }_{{{{{\rm{r}}}}}}=\tan {\theta }_{{{{{\rm{r}}}}}}$, while *q*_*θ*_ is a free fitting parameter that is found to decrease with the degree of confinement *d*/*b*. Namely, at a given angle the surface flow rate is lower for more confined, larger grains. When normalised by *q*_*θ*_, the data collapses, shown inset of 5(a).

Combining Eqs. (2), (3) provides an empirical model for the steady state surface flow rate in the silo as a function of silo grain flow rate, which is shown as solid lines in Fig. 5b, plotted with measured values as points. At low *Q*_g_, the surface flow rate is equal to that of the internal silo grain flow rate, represented by the dashed line. As *Q*_g_ increases, however, and the surface angle approaches its maximum ${\theta }_{\max }^{* }$, this parity breaks and the surface flow rate falls significantly below *Q*_g_. This effect results generally in a higher ${Q}_{{{{{\rm{s}}}}}}^{* }$ for larger *d*, in addition to the *Q* dependence.

### Central silo flow

We now wish to quantify the grain flow internally within the silo, below the grain surface, in more detail than previously discussed. For illustration, Fig. 4d shows the mean velocity in the central (*x* = 0) region ${\bar{v}}_{{{{{\rm{mid}}}}}}$, measured in a 1 cm^2^ moving square area below the tip of the descending grain surface, as a function of time. The gradual increase for *t* < *t*_c_ reflects the vertical grain velocity field inside the silo - the central velocity increases with proximity to the outlet. This can be described by the diffusion model in a fluid-driven silo:

4 $$v(x,y)=\frac{{q}_{{{{{\rm{g}}}}}}}{\sqrt{4\pi {B}_{\alpha }{y}^{\alpha }}}\exp \left(-\frac{{x}^{2}}{4{B}_{\alpha }{y}^{\alpha }}\right)$$

where *q*_g_ is the flux per unit depth across the gap, presently considered to be *q*_g_ = *Q*_g_/*ϕ*_s_*b* as transverse variation in the cell is minimal and the solid fraction is constant to leading order in the dense silo^18,29^; *b* is silo plate spacing and *ϕ*_s_ is the static solid fraction. Constants *α* = 0.7 and *B*_*α*_ = 1 ⋅ *d* m^(2−*α*)^ are henceforth taken as characteristic values observed in similar conditions^18^ to go beyond the earlier parabolic approximation. The grain flow rate in a central column of width *w* in the silo (as marked in Fig. 1c can be calculated by evaluating the integral of Equation (4) with respect to *x* between the limits $-\frac{w}{2}$ and $\frac{w}{2}$. It follows that this flow rate *Q*_tip_ can be written as a function of distance *y* from the outlet:

5 $$\begin{array}{rcl}{Q}_{{{{{\rm{tip}}}}}}&=&\frac{{\phi }_{{{{{\rm{s}}}}}}b{q}_{{{{{\rm{g}}}}}}}{2}{{{{\rm{erf}}}}}{\left.\left(\frac{x}{\sqrt{4{B}_{\alpha }{y}^{\alpha }}}\right)\right| }_{-\frac{w}{2}}^{\frac{w}{2}}\\ &=&{Q}_{{{{{\rm{g}}}}}}{{{{\rm{erf}}}}}\left(\frac{w}{4\sqrt{{B}_{\alpha }{y}^{\alpha }}}\right)\end{array}$$

### Internal-surface flow mass balance

In this viscously unstable system, the gravitational surface flow is acting to stabilise the grain surface. In order to maintain this stability, the surface flow rate ${Q}_{{{{{\rm{s}}}}}}^{* }$ must be sufficient to supply the flow *Q*_tip_ demanded locally below it in the centre of the silo. The lowest height *y*_c_ at which this can still be achieved is therefore when ${Q}_{{{{{\rm{tip}}}}}}={Q}_{{{{{\rm{s}}}}}}^{* }$. Using Eqs. (3), (5), this is solved numerically for *y*_c_ and plotted as a function of imposed flow rate *Q* in Fig. 3a, with *w*/2 = 7 mm as marked on Fig. 1e, equal to approximately three standard deviations of a typical wormhole velocity profile. This model correctly predicts that wormholes form higher in the silo for both higher *Q* and smaller *d*, and shows quantitative agreement with measured *y*_c_. For *y* < *y*_c_, where ${Q}_{{{{{\rm{tip}}}}}} > {Q}_{{{{{\rm{s}}}}}}^{* }$ the disparity in grain flow is appropriated by fluid, causing dilation of the packing and the formation of a wormhole. As *Q* increases further, the upper grain surface becomes increasingly unstable, resulting in a transitional region in which fingers begin to form before eroding into wormholes while the silo rapidly empties, precluding the existence of a steady-state in which Eqs. (4), (5) hold.

### Phases of behaviour

**Fig. 6:**
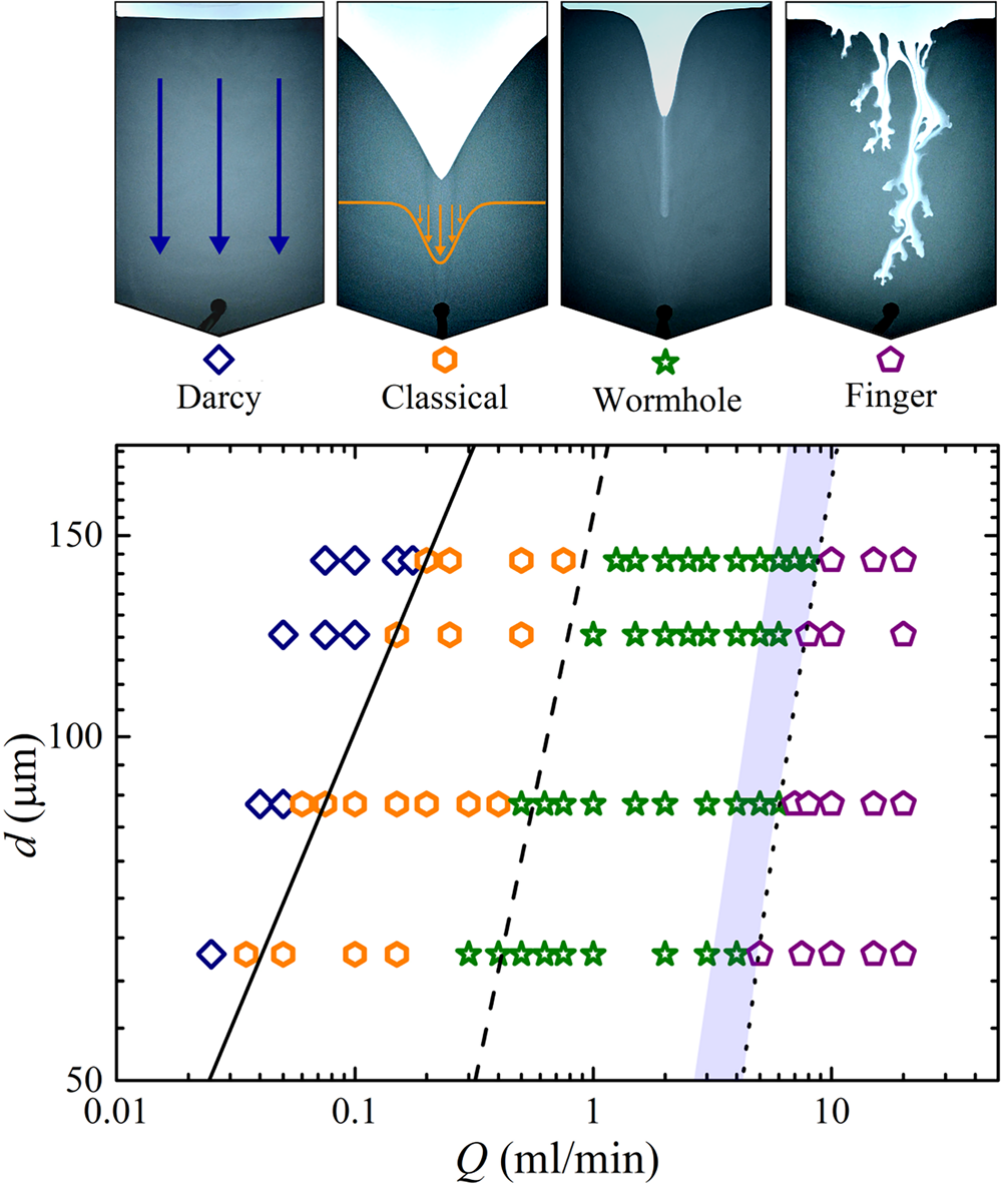
Phase diagram of silo behaviour. Each of the four behavioural regimes observed in experiments are illustrated at the top of the figure. In the graph below they are represented in terms of total flow rate *Q* and grain size *d*, where the Darcy flow (blue diamond), classical silo flow (yellow hexagon), wormhole (green star) and viscous finger (purple pentagon) regimes are marked in *Q* − *d* space. For wormhole and viscous finger transitions, a continuous line was obtained via linear fits (*R*^2^ > 0.96) of the calculated numerical transitions for each grain size. The shaded area represents an intermediate region between wormhole and viscous fingering.

A phase diagram constructed from experiments is plotted in Fig. 6 that maps the various behaviours observed in the controlled parameter space of *d* and *Q*. This shows Darcy flow at low flow rates where the fluid flow is insufficient to mobilise the grains in the horizontal outlet, and a transition to classical silo is flow marked (solid line) by the flow threshold ${Q}_{{{{{\rm{m}}}}}}=\frac{\mu \Delta \rho gk{A}_{{{{{\rm{o}}}}}}}{\eta }$ where *k* is the permeability using the Kozeny-Carman equation, *A*_o_ is the outlet cross sectional area, and *μ* is grain friction coefficient taken nominally here as $\tan (3{3}^{\circ })$. This considers when the pressure due to Darcy’s law exceeds the gravitational friction of the grains in the outlet^10,30^. This behaviour is therefore specific to systems with a long, laterally oriented outlet and would not occur with an outlet of sufficient size positioned on the bottom^4^. Beyond this threshold, classical silo flow occurs until a further transition to the wormhole regime, marked (dashed line) by solving ${Q}_{{{{{\rm{s}}}}}}^{* }={Q}_{{{{{\rm{tip}}}}}}$ numerically for the flow rate at which *y*_c_ = *w*/2, and the distance from the interface to the outlet is comparable to the wormhole width. More generally, system confinement is likely to influence wormhole onset due to the important role of the sidewalls. As the grain flow rate in confined heaps has a strong dependence on wall gap ${Q}_{{{{{\rm{s}}}}}}^{* } \sim {b}^{5/2}$ for a given angle^31^, a less confined silo may require comparatively faster imposed flow before the criteria ${Q}_{{{{{\rm{tip}}}}}} > {Q}_{{{{{\rm{s}}}}}}^{* }$ is met to achieve wormhole formation.

At higher *Q*, a transition to finger patterning is observed. In contrast to horizontal fluid-driven fingering in granular media that exhibits pressure-related regimes^11^, the gravitational surface grain flow acts to smooth initial finger instabilities, delaying their onset. Therefore only when $Q\,\gg \,{Q}_{{{{{\rm{s}}}}}}^{* }$, and the characteristic finger velocity is much faster than the falling grains, can a finger-like instability persist through the silo to the outlet without morphing into a wormhole or stabilising to classical flow. As a guide to mark this transition to fingering in Fig. 6, a dotted line represents the relationship $Q\propto {Q}_{{{{{\rm{s}}}}}}^{* }({\theta }^{* }={\theta }_{\max }^{* })$. This considers when the imposed flow rate reaches some threshold proportional to the maximum surface flow rate as described by the empirical model; the dotted line represents when *Q* is eight times that of the ${Q}_{{{{{\rm{s}}}}}}^{* }$ plateau.

## Conclusions

In summary, an array of behaviours has been observed in a fluid-driven granular silo, including the formation of wormholes, in which all grain flow occurs in a rapid central channel. The instability may be generalised as a competition between gravitational and viscous effects, and was found to occur following an imbalance between the grain flow drawn by central silo region, and that avalanching towards it at the free surface. If the imposed flow rate is high enough, the surface flow is insufficient to placate the demand of the silo below and water takes the place of the absent grains. Empirical models were formulated for surface and internal flow and used to predict the height in the silo at which the wormholes form, finding agreement with observations at different flow rates and grain sizes. The behaviour was contextualised with a phase diagram marking the transitions between the different regimes. The granular suspensions used here comprise materials that are ubiquitous in nature and industry; the incidence of wormholes in real-world submerged granular systems could lead to significant local variation in material fluxes, particularly in confined geometries such as fault gouge and fractures.

Future work may entail the development of an accurate rheological model of submerged surface grain flow, coupled with the internal silo flow field to correctly predict inclination angle and subsequent surface grain flux. The role of sidewall friction may be particularly important in this respect and a potential factor in the onset of wormhole formation. Experiments in silos with larger internal spacing could prove useful to assess this practically.

## Methods

Experiments were performed in a manner similar to previously detailed work considering submerged silos^18^. A quasi-two-dimensional silo of width 20 cm, height 30 cm, and internal spacing *b* = 0.05 cm, was constructed from two glass plates separated by double-sided tape along the edges and base. It had an open top edge and a horizontally oriented 4 mm outlet hole drilled through the front plate at the base. Initially, the cell was filled with water before adding soda lime glass beads (density 2.47 g/cm^3^) from the top, allowing them to settle under gravity to form a bed with a horizontal top surface. The grain diameters, given in Table 1, and narrow plate spacing result in a highly confined system such that 4 ≲ *b*/*d* ≲ 8, though no clogging or intermittency were observed. A syringe pump (Harvard PHD Ultra) at the outlet was used to withdraw a total volume flow rate *Q*, while a second pump replenished water at the top to maintain a constant hydraulic head. The silo was backlit with an LED screen and flow was captured with photographic equipment. Critical wormhole formation height *y*_c_ was determined visually by identifying the earliest captured frame in which a wormhole was evident and measuring the distance from the outlet to the point of formation; *t*_c_ corresponds to the time at which this measurement was taken. Free surface angle *θ* was calculated by a linear fit of the surface profile at its steepest and concurrently straightest. θ^*^ is the time-average of *θ* at its maximum plateau (see Fig. 4b). Particle image velocimetry (PIV) was carried out using PIVLab toolbox in MATLAB^32^ to extract grain velocity fields. Local solid volume fraction *ϕ* measurements were obtained by mapping transmitted light intensity *I* according to a Beer-Lambert relationship $I={I}_{0}\exp (-\epsilon b\phi )$ where *I*_0_ is the intensity with no grains present (*ϕ* = 0) and *ϵ* is a parameter related to the absorption and scattering of light through the granular media, determined considering *I* for a static bed where *ϕ* = *ϕ*_s_^30^. Free surface grain flow rates down the avalanching slopes were calculated by ${Q}_{{{{{\rm{s}}}}}}=2b\int\phi ({x}^{{\prime} }){v}_{{y}^{{\prime} }}({x}^{{\prime} })d{x}^{{\prime} }$, where ${x}^{{\prime} }$ and ${y}^{{\prime} }$ are the dimensions perpendicular and parallel to the surface flow, respectively and the factor 2 accounts for both slopes. The integral was taken by the trapezoidal method from the free surface to where the velocity decayed to two orders of magnitude below its maximum, or a limit of 1 cm below the surface in the minimal occurrences otherwise. Images of grain depletion in the silo were used to measure the total grain flow rate *Q*_g_ for each experiment, assuming the solid fraction in the silo to be *ϕ*_s_^30^. Above *Q* = *Q*_m_, *Q*_g_ increases linearly with *Q* for a given grain size; linear fits were used to plot the model *y*_c_(*Q*) in Fig. 3. Static solid fraction was calculated by measuring the packing volume taken up by a known mass of grains deposited in the cell and is displayed in Table 1 for each grain size, in addition to the angle of repose measured in situ within the silo. These quantities generally reflect the increasing confinement^33,34^.

## Supplementary information

Description of Additional Supplementary Files

Supplementary Movie 1

Supplementary Movie 2

## Data Availability

Experimental data is available on the Zenodo data repository 10.5281/zenodo.17227173^35^.
