# Supplementary material for: Wormhole formation in fluid-driven granular flow
Source: Commun Phys. 2025 Nov 24;8(1):468. doi: 10.1038/s42005-025-02366-w (PMC12646110; doi:10.1038/s42005-025-02366-w)
Supplement: Supplementary file 1 — Description of Additional Supplementary Files [file 42005_2025_2366_MOESM1_ESM.pdf]

Description of Additional Supplementary Files

File name- Supplementary Movie 1 –

File description- Video capture of wormhole formation in 75-100 µm grains at 2 ml/min.

File name- Supplementary Movie 2 –

File description- Video capture of viscous fingers in 53-75 µm grains at 20 ml/min.
